# An ethnographic study to develop a taxonomy of lies for communicating with people with moderate to severe dementia

**DOI:** 10.1177/09697330241246087

**Published:** 2024-06-04

**Authors:** Jane Murray, Juliana Thompson, Michael Hill, Ian James

**Affiliations:** 5995 Northumbria University; 5995 Northumbria University; 5995 Northumbria University; 5633 CNTW NHS Foundation Trust

**Keywords:** Dementia, ethnography, lie, Lie affective reflective model, taxonomy, truth

## Abstract

**Background:**

There is no definition of what constitutes a lie when working with people with moderate to severe dementia. Lies are often defined as therapeutic with no evidence of how therapeutic value is gauged. There is no previous research that observes lies being told or the impact the lies have on people with dementia.

**Aim:**

The aim was to develop a taxonomy of lies for use when supporting people with moderate to severe dementia and then use this to develop a model, which could be used to explore the impact of lie telling.

**Research design:**

Ethnography was used, with the researcher observing and recording lies told to people with moderate to severe dementia, by professional caregivers. Data analysis was based on Burnard’s thematic analysis.

**Ethical consideration:**

Ethical approval for the study was obtained from Northumbria University, the Research Ethics Committee (REC) and the Health Research Authority (HRA) in the United Kingdom. Study number 227508.

**Findings:**

Six categories of lie were identified; blatant, avoidance, familiarity, props, banter and going along with. Spontaneous and planned lies were observed. Motivation, validation and genuineness were key in terms of how the person with dementia responded. These affective domains were used to develop the Lie ARM (affective reflective model).

**Discussion:**

Planned and spontaneous lies are an effective intervention for people with moderate to severe dementia. Healthcare professionals need to be aware of their motivation for using lies, as well as the impact they have on patients.

**Conclusion:**

The taxonomy is an objective way to categorise lies. It supports increased reflection, particularly if used in conjunction with the Lie ARM. The Lie ARM is a useful tool to help staff reflect on practice or to predict whether a proposed lie is likely to be therapeutic.

## Introduction

This study aimed to develop a definitive taxonomy of lie telling in relation to people with dementia that would bring a level of definition and objectivity to the subject. It is a way of classifying things into related groups, based on common factors, rather than allocating a level or point on a scale. It is important that professional carers can identify and then define the lies that they tell, in order to be able to reflect on them effectively. Reflection on action and in action^1^ has been identified as an essential skill for healthcare professionals. It helps them to develop good practice and analyse the impact of their actions. Reflection is most effective when a structured model is used and the starting point for all models is identifying the topic or element to be reflected on. The Lie ARM (affective reflective model) was then developed to support structured reflection focused on when professional caregivers tell lies to people with moderate to severe dementia.

## Background

Lying during the care of people with dementia is a common if controversial practice^2^ which presents a complex dilemma for professional caregivers. The difficulty is that telling the truth to patients with dementia can often cause major distress or do certain harm,^3^ for example, when a person with dementia is asking after a deceased relative. Hence, many professional care staff admit to telling lies when delivering care,^4^ even if it means contravening their codes of practice.^5,6^

Despite the huge weight attached to the word ‘lie’, it is difficult to find a clear definition in the literature in relation to people with dementia. An early study by Blum^7^ examined deceptive practices conducted by family carers looking after people with dementia at home. As part of this 4-year study, Blum looked at how families define deception, how deceptive practices are learned and what forms the deception takes, such as collusion with others and two-party deception. This resulted in a range of terms being identified, for example, not telling, going along with, little white lies and tricks, that are used to describe deception. Blum concluded that the use of deception is both routine and contextualised. It is primarily used for informal social control. The limitation of this study is that caregivers were asked about their perceptions of what had been said or done, rather than being observed.

*What is Truth? An Inquiry about Truth and Lying in Dementia Care from the Mental Health Foundation*^8^ investigated if, why, when and how non-truth telling is justified. Throughout the document, a range of terms are used including truth, lying, deception and therapeutic lies. None of these terms are clearly defined. The closest the report comes to defining lying is when it concludes that lying is ‘where a carer initiates purposeful deception’ (8, p.147). This definition of lying can be challenged as not all lies involve deception.^9^ There are occasions where both parties engage in lie telling and are fully aware that the truth is not being told, therefore, no one is being deceived. The report concludes that whilst telling the truth must always be the starting point, it may not be in a person’s best interest, to continue to repeat that truth. In some instances, in order to reduce distress, it may be beneficial to consider a continuum from whole truth telling to lying, with lying being used as a last resort. What was repeated throughout the report was the significance of intent in relation to telling a lie, and the inquiry only focused on instances where lies would be told with best intent, whilst acknowledging that this may not always be the case.

In other studies, participants identified that they only told therapeutic lies, and they were used in order to meet the needs of the patient.^2^ These studies were all carried out retrospectively, where participants were asked to recall the lies they had told and why, rather than observed. Therapeutic lies are not defined in the literature but are implicit in that they are lies told with the intention of having a positive effect on the receiver and are told with the intent or motivation to reduce distress. As previous studies use self-report from participants, this is hard to verify. The ethnographic nature of the study reported in this paper meant that the actual lies told were observed, documented and contextualised so that what was said could be verified and then clarified with the teller giving insight into the motivation and observable impact of each communication.

One of the only taxonomies available in relation to telling lies to people with dementia was published in 2017.^10^ It cites Blum,^7^ Vrij^11^ and several others in terms of their individual definitions of a range of alternative words and phrases that are used to describe and define lie telling. The difficulty is that there is no consensus around each author’s definition, many of which are context-specific and not necessarily related to clinical practice.

## Methodology

### Study design

This study is unique in the field of lie telling to people with dementia, in that it used focussed or rapid ethnography as a methodology.^12^ It is also sometimes referred to as micro ethnography^13^ or selective intermittent ethnography.^14^ This ethnography was focused specifically on communicative interactions between professional caregivers and people with moderate to severe dementia. It has the advantage of providing an in-depth depiction of a single interaction, rather than trying to portray an entire scene or context which could potentially reduce the depth or detail of the study.^15^ Intermittent ethnography reflected the fact the researcher would visit the wards where the data was to be collected frequently and in short bursts, with most shifts lasting approximately 8 h, rather than the researcher residing within a community. The researcher’s shift pattern reflected that of the rest of the staff team. The lead researcher was an overt participant observer who worked as part of the ward team as a band five staff nurse, to observe and record lies that were told during each shift. The lead researcher had an intimate knowledge of the field being studied, which was highlighted by Knoblauch^16^ as being particularly effective for studying communicative activities or experiences by communication.

### Participants

Active participants in the study were any professional caregivers, working into two wards that took part in the study, who were observed telling lies to patients. Lies were defined as anything that was not the whole truth. The wards were based in hospitals in the Northeast of England, with a remit to meet the needs of people with moderate to severe dementia. The participants could be a regular, ward-based team member or someone who visited the area, such as a physiotherapist or pharmacist.

Patients in the area were considered as passive participants. They all had moderate to severe dementia and impaired short-term memory, which limited their capacity to consent to participate in the study. In the UK, people who lack capacity are still able to participate in research, as long as it is conducted within the parameters of the Mental Capacity Act (MCA).^17^ Under the auspice of the MCA, consultees of all patients were given an information pack and asked for an opinion as to whether their relative or friend should participate in the study. All active participants and consultees agreed to participation in the study.

### Ethical considerations

From the beginning of this study, it was always anticipated that there would be some ethical challenges, even though it met the criteria for approval of research involving adults who lack mental capacity.^17^ Generally, the challenges were not related to the activity of the study, but predominantly caused by the wording of it; to undertake a critical analysis of the concept of lying in clinical practice, in the context of people with dementia. As previously highlighted, the word lie tends to evoke strong and usually negative emotions in people.^18^ There are other terms that perhaps soften the emotional impact such as white lie or fiblet but, after much discussion with the research team, it was felt that these words did not effectively identify what the study intended to do and could potentially be construed as deceptive in themselves. Concerns were also raised about a study that would potentially show that health professionals tell lies to patients. Eventually, the study gained full ethical approval from Northumbria University, the NHS Research Ethics Committee REC and the Health Research Authority (HRA) study number 227508.

### Data collection

Data were collected across the two sites over a period of 4 months which equated to 338 hours of observation time. 250 lies or untruths were recorded during this period from 63 participants. For data collection purposes, anything that was not the whole truth was considered a lie. In some cases, interactions were documented and the validity of them in terms of truthfulness had to be verified later in the shift. The researcher documented interactions in a notebook either contemporaneously when appropriate or as soon as possible after the interaction had taken place. This was often followed by informal or conversational interviews to check with participants that they agreed with what had been recorded and the context in which it had been said.^19^ It was important to do this as soon as possible after an item was recorded, so that the correct participant could be identified (no information was kept that would enable a participant to be identified at a later stage) and that their memory of the interaction was still current. This type of respondent validation was important in terms of error reduction and improving accuracy.

### Data analysis

The data analysis was conducted using a combination of methods which could be described as a method of thematic content analysis. The process followed a similar path to that of analytic induction which was originally developed by Znaniecki in 1934^20^ and further refined by Hammersley,^21^ in relation to ethnography. The aim was to identify common themes that emerged across the interactions; therefore, the formulate/reformulate hypotheses stage was where the categories or themes were extrapolated, more in line with Burnard’s method of analysing transcripts,^22^ although only the early stages of this model were mirrored. During this process, it was identified that some lies spanned more than one category. After reconsidering the themes, it became apparent that where data spanned more than one category, there was still a dominance that related to one theme more strongly than the others. In these instances, the data was categorised according to the strongest theme. To support this process, notes from the researcher’s daily reflections were added to each piece of data. This formed part of the emergent analysis which is significant in analytical ethnography.^23^

## Results

### Taxonomy of lies

**Figure 1.**
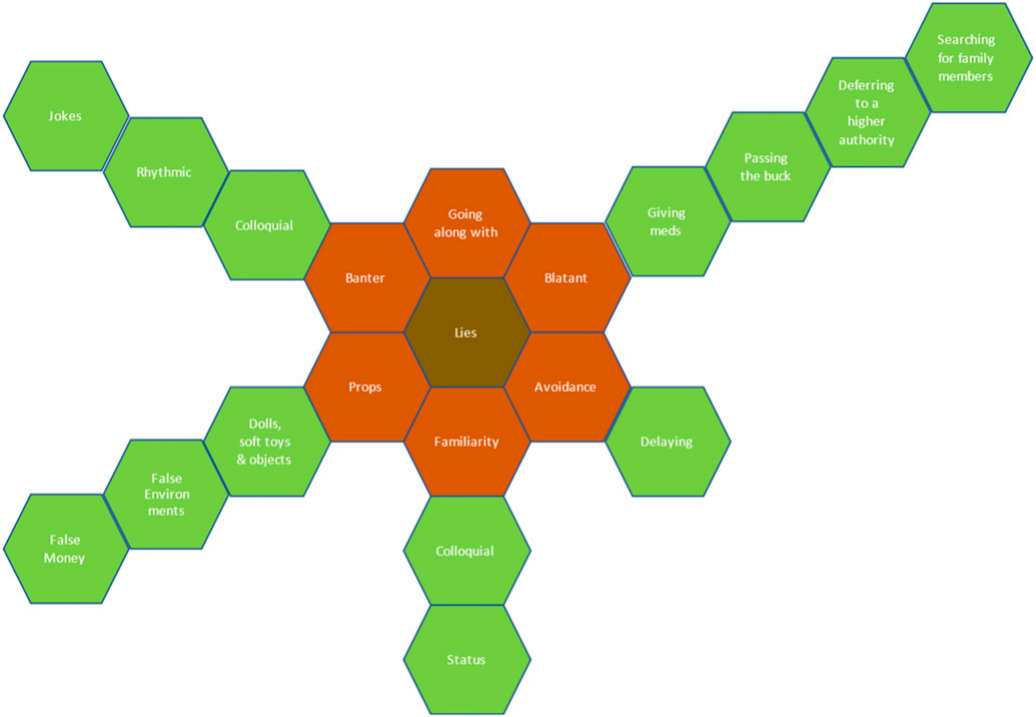
The taxonomy of lies.

The Taxonomy of Lies is based around 6 key categories. The categories were generated on the assumption of the healthcare professional’s truth, rather than that of the person with dementia, as the healthcare professional’s truth is more likely to be shared by those around the person with dementia. It is important to acknowledge that the development of the taxonomy was not done in vacuo but evolved from extensive reflexivity and detailed examination of the observational field notes and daily reflections.^24^ When the taxonomy is viewed in the form of the model, it is important that this represents a visual guide that is fluid Figure 1.

Central to the taxonomy is lies. In terms of this study, everything that is not the wholetruth is deemed to be a lie and would fit in the central block. The placing of the categories on the model is arbitrary as no category has more significance or priority over another. Some lies told will fit into multiple categories, but in terms of classification, there was always one element of the untruthful part of the interaction that was more prominent, and the lie was categorised in terms of the largest or most significant element. For example, in props when discussing the use of soft toys there was quite often an element of going along with the patient’s reality, but the main focus of the lie was around the use of props, hence its theme and subsequent sub-theme. All the data collected during this study fits into one of the six key categories and may then have been allocated to a subgroup.

### Blatant

In the simplest form, a blatant lie is an untruthful communication, which if successful, will deceive the receiver (remembering that deception only occurs when the lie is believed by the receiver). It is blatant because it is simply a lie, initiated without prompting, by the teller. It is generally given as a definitive and correct statement, even though the teller knows it to be untrue.

Four significant sub-themes that were identified within the central theme of blatant: medication administration, passing the buck, deferring to a higher authority and searching for family members.

This is an example of a blatant lie around medication administration;

Nurse: ‘Here’s a little drink for you’ (paracetamol).

Patient: ‘Oh. Oh’ (puts paracetamol to lips).

Nurse: ‘It’s nice. Have a little drink’.

The patient accepted the medication under the guise of ‘a little drink’. At no point was the patient told it was medication. The nurse was very genuine and kind in her communication and her motivation was clearly to meet the needs of the patient, hence the medication being accepted.

This type of blatant lie, particularly in relation to people with dementia, presents some ethical challenges. Effectively, the paracetamol was being given covertly. The patient was offered a drink of an unidentified substance. The nurse knew that if she was honest about the drink the patient may not take it, so gave it covertly as ‘a little drink’.

Passing the buck was also evident around administering medication;

Nurse: ‘Here’s your painkillers, open your mouth’.

The patient responded verbally but not coherently. However, she also made it plain that she did not want to take them.

Nurse: ‘It’s for your knee. Your daughter says you have to have them’.

Patient: ‘No’.

The nurse gave a big sigh and dropped her shoulders, clearly conveying her irritation.

Nurse: ‘Your daughter rang and said you have to have them’.

This was said in a very firm voice. The patient then allowed nurse to put tablets in her mouth. The patient then spat them out later. This was a frequent habit of the patient, so a member of staff had been allocated to observe the patient for a period of time to see if she either swallowed them or spat them out.

The interaction was brusque, and the nurse appeared tense as she approached the patient. It is very easy to approach the task negatively with a patient who is often challenging, but that approach tends to communicate anxiety to the patient, increasing the chances of them refusing.^25^ The nurse interacted with the patient in a very matter of fact tone and lacked any warmth or empathy. The motivation of the nurse was to complete the medication round, not to meet the needs of the patient, consequently, telling a lie did not help to get the patient to take her medication. Equally, lying did not have a negative impact either since, once the medication had been spat out, the patient had forgotten the interaction within minutes. On another occasion, two nurses entered into a deceitful communication that involved deferring to a higher authority, which was again ineffective due to lack of genuineness and the nurse being motivated to meet her own needs rather than those of the patient:

It was morning and the patient had been agitated since coming out of their bedroom. He had been sitting in lounge, shouting and swearing intermittently. Two nurses approached him with his medication.

Patient: ‘What are they?’

Nurse 1: ‘Your tablets’.

Patient: ‘What are they for?’

Nurse 1: ‘All sorts of things’.

{This initial untruth was avoidance as she did not want to tell him that it included anti-depressant and anti-psychotic medication}

Nurse 2: ‘You need to take them, or the doctor will tell me I’m not doing my job’.

Nurse 1: ‘The doctor says you have to have them’.

Nurse 2: ‘I’ve had mine’.

Patient: ‘What? All of them?’

{This was said as the patient looked into a medicine pot with several tablets in}

Nurse 2: ‘Yes. They make you feel better’.

Patient did not take any of the tablets and the frustration of the nurses was evident.

Blatant lies were the biggest overall theme recorded, yet none of the lies were care planned, they were all spontaneous. Spontaneous or unconscious lying has not previously been identified in the literature as earlier research relied on the accounts of carers (familial and professional) after the intervention and suggested that decisions about lie telling and deception were conscious interactions triggered by specific dilemmas, often related to confidentiality and influencing behaviour.^26^ In the following example, a blatant ‘searching for family members’ lie was told by a HCA:

Patient: ‘Mam. Mam. Where’s me mam?’

Healthcare assistant: ‘She’s at home. She’s fine’.

Patient: ‘Are you sure?’

Healthcare assistant: ‘Yes she’s at home’.

Patient: ‘Oh’.

The healthcare assistant spoke in a confident and reassuring manner. The reassurance conveyed by the tone of the healthcare assistant placated the patient who seemed satisfied by the answer and did not ask again.

This is the most varied theme in terms of response from the patient. The response from the patient was completely reliant on how the lie was said and the motivation of the teller for expressing it, rather than the actual content.

### Familiarity

Familiarity is where a word or phrase is used that is known or familiar to the receiver. It can be used consciously or unconsciously and often serves as a social lubricant.

Colloquial and status were two clearly identified sub-themes. Colloquial familiarity stands out because it is spontaneous, and the only theme where the communication is specific to the teller rather than the receiver. Staff who used colloquial and familial terms, tended to as part of their daily discourse, regardless of who they were speaking to. Colloquial familiarity was never observed to have a negative impact on the receiver. Such colloquial phrases would be considered to be part of the local idiom and, in some areas of the country, like the Northeast, are far more acceptable than in others.^27^

Healthcare assistant: ‘Hello pet. Shall we get your horses on later?’

Patient: Did not respond.

The use of the term ‘pet’ made no difference at all to the patient’s response and was more specific to the healthcare assistant than the patient.

Status familiarity was when familiar terms was used specifically and consciously, usually as part of planned or accepted care. These are quite separate in terms of the motivation for the teller using them, for example when a particular term or title was conveyed on a patient that may have been true in the past or in a different context but was not true for their current situation. In these cases, the familiar term was used consciously and was specific to each patient, as illustrated below:

A healthcare assistant approaches a patient to take him for breakfast.

Healthcare assistant: ‘Morning Boss. Come and get some breakfast’.

Patient goes with healthcare assistant.

The patient had been a Captain / chief engineer on a ship for many years. He could be difficult to engage with but sometimes, calling him Boss helped as this is what he had been called throughout his working life. He responded well to being given that extra level of respect and then took on the mantle or behaviour of a ‘boss’. The use of the term ‘Boss’ was care planned and used consistently by all staff. This is more of an untruth / lie than some of the other terms in this category if it is considered in the context of the healthcare professional’s truth. However, in the context of the patient’s reality, it was the truth, as he still believed himself to be ‘the Boss’.

### Props

The word props in the study identifies that the healthcare professional had used an aid or object to support or reinforce communication with the person with dementia. It was always an inanimate object to which certain, although not always truthful, properties were assigned. In all cases, the prop was something that the patient would recognise from earlier in their life and would have made sense to them in the context of their previous roles or relationships. The props used ranged from soft toys being treated as live animals through to the use of fake money which happened frequently on both wards. There was also the use of non-alcoholic beers and drinks and a bar set up on one of the wards. The patient was often unaware that the relationship they were engaging with or about, had long ceased to exist in other people’s eyes. Most instances where props were used to support lie telling were care planned.

Patient (male) in a very aggressive tone: ‘Where’s my money?’ (throws table)

Healthcare assistant: ‘I’ll go and get your money. It’s in the safe’.

Patient, shouting: ‘Where’s my money?’

Healthcare assistant 2: ‘(Healthcare assistant 1) has gone to get your money’.

Healthcare assistant 1 returns with fake money and hands it to patient who snatches it from her.

The patient then put the fake money in his trousers, becoming less angry and begun walking around the ward in a calmer fashion.

Occasionally props were used spontaneously with good effect.

A male patient kept walking into a ward visitor, making physical contact.

Healthcare assistant: ‘C’mon [patient’s name], help me do this paperwork (showing patient the observation file) then jobs a good un’.

Patient follows

Healthcare assistant: ‘You sit there and help me fill these forms in’.

Patient took some blank forms and sat moving them around, completely engaged in the process.

There appeared to be genuine investment in the use of props by staff, and they were always used with the purpose of encouraging positive social interaction or validating the patients’ feelings, which was reflected in the consistently positive results observed.

### Banter

Banter is a mode of conversation that is essential in building and maintaining relationships.^28^ It allows people to belong or to be part of a group which has a shared understanding of a particular event or interaction.^29^ Banter is about spontaneity, often with short responses that follow a pattern of conversation that is likely to be well established in a person’s long-term memory. It may be based on truth but is more likely to be underpinned by a lie.^30^ For many people, it is an essential, appreciated and understood part of usual conversation that often enhances social cohesion and is often regarded as a pleasurable activity between two or more parties.^31^

It is the only theme in which both the teller and receiver were fully aware that the content of the interaction was untruthful, but both were in collusion and happy to continue with the interaction. Whilst the interaction is based on untruths, it is lying without deception as both parties were wilfully engaging in a conversation which they did not believe to be true. It is the only form of lying which is not underpinned by deceit. It is subdivided into two subcategories; colloquial banter and rhythmic banter.

### Colloquial banter

In this theme, the conversation followed a well-established path, even in terms of content. It used familiar, often colloquial phrases or references. This seemed to create a feeling of safety or confidence in terms of the person with dementia feeling able to respond immediately and appropriately to what was said. The example below was witnessed on both wards which highlights the frequency and generality of some well-established sayings:

Patient turns to other patients in the lounge: ‘Goodbye everyone’ and waves, heading for the door.

Nurse: ‘See you later alligator’.

Patient laughs and continues walking around the ward.

This is a well-used local phrase and there was immediate recognition from the patient.

Whilst no one actual believes what the healthcare assistant said was true, it provided a positive and effective platform to interact with patient while the healthcare assistant tried to carry out what was in fact, quite a difficult job.

### Rhythmic banter

The second sub-theme of banter identified was where a conversation followed a specific pattern or rhythm without the actual content being previously established. It was generally a short and sometimes humorous exchange. Patients seem to engage easily and fluidly in this type of discourse, perhaps because there was no pressure to tell the truth or be right. The sole identified purpose of the communication was to interact on a social level, as seen in the observation below:

A patient was in the sitting room with a couple of members of staff. Everyone was sitting down having a mid-morning drink.

Patient: ‘What is your job today?’

Healthcare assistant: ‘My job today is to sit in here with you lovely young ladies’.

Patient: ‘Hahahaha. I wish. Young ladies? As if?’

This statement had clearly amused the patient who immediately identified the untruth, and both parties were fully aware that the female patients in the room were not young.

Banter is a standalone theme of social interactions based on lies. It relies on both parties engaging with a shared truth or more often shared lie. It does not involve deception. Both parties are likely to have used or heard the phrases used over many years, making them familiar and comfortable, with expected outcomes. There is less emphasis placed on content than there is on patterns of speech and outcomes. Some spontaneous banter can occur which uses new phonology but still has a known rhythm and pattern in which both parties engage. The motivation of staff to engage positively with patients in a more friendly and less clinical manner was evident, as was the pleasure derived from patients during these interactions. The genuineness of staff when engaging with banter is also evident. It was observed to be a pleasurable experience for both parties in most instances. This theme of shared lies has not previously been identified by other studies.

### Going along with

Most commentators identify going along with as either not lying or not as extreme or unpleasant as blatant lying.^7^ To go along with someone is to join them in their reality that they are already in, but this may mean leaving our own reality or truth. The strategy of going along with a patient was observed many times and on a range of levels. Sometimes it would involve quite structured or complex dialogue. The following interaction took place between an agitated female patient and a healthcare assistant. The patient regularly threatens staff with legal action but cannot understand that she is detained under Section 3 MHA.^32^

Patient: ‘My friend is coming later. He’s from Leeds. He’s a barrister’.

Healthcare assistant: ‘Well when he gets here, we’ll make him a cup of tea. Leeds is a long way’.

If the healthcare assistant had corrected the patient using her reality, it is likely to have led to conflict and distress. By going along with the patient’s truth, the interaction remained positive.

Many observations of going along with were much shorter and validation of the patients’ emotions were key. Often the patients had limited verbal skills and were difficult to understand and the following three phrases were observed to be effective.

‘Oh right. I see’.

‘OK, yes, it will be definitely be fine’.

Nodding, ‘yes, I’m sure it will be’.

Staff were agreeing with patients and reassuring them even though they had not understood what the patient had said. They empathised and then validated the emotion which reduced the significance of what was actually said. In these instances, the professional was engaging with the reality of the patient and going along with them. However, the communication cannot be wholly truthful for both parties. One party (in this case, the professional) is working outside of their own truth and therefore could be said to be lying. Going along with was an important and positive intervention.

### Avoidance and (delaying)

The study defined avoidance as not wanting to cause upset by confronting people with the truth. A frequent example was when a patient was looking for a relative, who was often deceased.

Patient: ‘Where’s wife?’

Nurse: ‘I don’t know. I haven’t seen her’.

Patient: ‘I need to speak to her’.

Nurse: ‘As soon as I come across her I will let you know’.

The nurse spoke kindly and confidently, as if they would most definitely come across the patient’s wife, although this was not going to happen. The patient walked away, satisfied with the response. The nurse had avoided saying that his wife was dead but had engaged in an alternative form of lie telling as the wife was deceased.

Delaying often used some form of ‘I’ll be back in…’ It was often teller specific rather than considering who the receiver was. It was observed across all staff disciples.

Patient: ‘Excuse me? Excuse me?’ (beckoning across sitting room).

Pharmacist: ‘Hi (patient) I’ll be back in 5 minutes. I just need to go and do something’.

Patient: ‘I need some help’.

Pharmacist: ‘I will be 2 minutes. You know I always come back and see you’.

Pharmacist left the ward without returning to the patient.

On one occasion, delaying was also observed to have a negative effect, which was rare. However, the negative reaction was observed to be short lived (less than a minute). This was due to the patient rapidly forgetting the interaction due to their severely impaired short-term memory.

Patient: ‘Can I be out?’

Healthcare: ‘Not at the minute’.

Patient (shouting): ‘Not at the minute. I’ve been here since 1985’.

Healthcare: ‘Ditto’.

Patient: ‘Yes but you are paid to be here and I am just locked up’.

The healthcare was not particular engaged with the patient during the interaction and made no attempt to validate the patient’s emotions. This served to highlight the significance of motivation and validation in relation to the success or otherwise, of an interaction.

## New knowledge – spontaneous lies

A major finding of this study was that professional care givers spontaneously lie, on a regular basis, without real purpose or motivation. At times, participants were observed to respond to a patient with a lie in an almost automatic or unconscious way, with no real intent other than responding to the patient in some way or disengaging from the conversation. On these occasions there was little emotion attached to the professional care givers’ responses. Sometimes they were effective but at other times they were responded to with indifference. In all themes there were examples of spontaneous lying. The example below is from ‘going along with’.

Patient (shouting): ‘Bread bread get my bread’.

Healthcare assistant: ‘OK. It’s in the fridge. I will go and get it now’.

The healthcare assistant walked out of one door and came back through another, sitting down in a different chair. As soon as the healthcare assistant was out of sight, the patient stopped shouting. There was no recognition when they came back into the room. The lie did meet the patient’s needs in that they stopped shouting so it could be assumed that they were less distressed, but there did not appear to be any other real impact. The lie was spontaneous but effective.

### The Lie ARM

The affective domains of validation, genuineness and motivation have reoccurred throughout the research. The impact they had on interactions was apparent during every interaction regardless of the lie being told and were the main influencers with regard to patient outcomes. Each of these themes are covered to varying degrees in the literature but have not previously been explored in combination when considering lie-telling.

Many of the lies that were witnessed could be considered or classified as therapeutic. That is, they met the needs of the patient in some way, such as lowering distress, maintaining a relationship or supporting a level of social interaction. However, some lies had no observable impact and in one case, caused short-term irritation for the receiver. After observing such a wide range of untruthful interactions, it has become clear that the key elements of whether a lie results in a positive interaction for the patient is how and why it is said.

**Figure 2.**
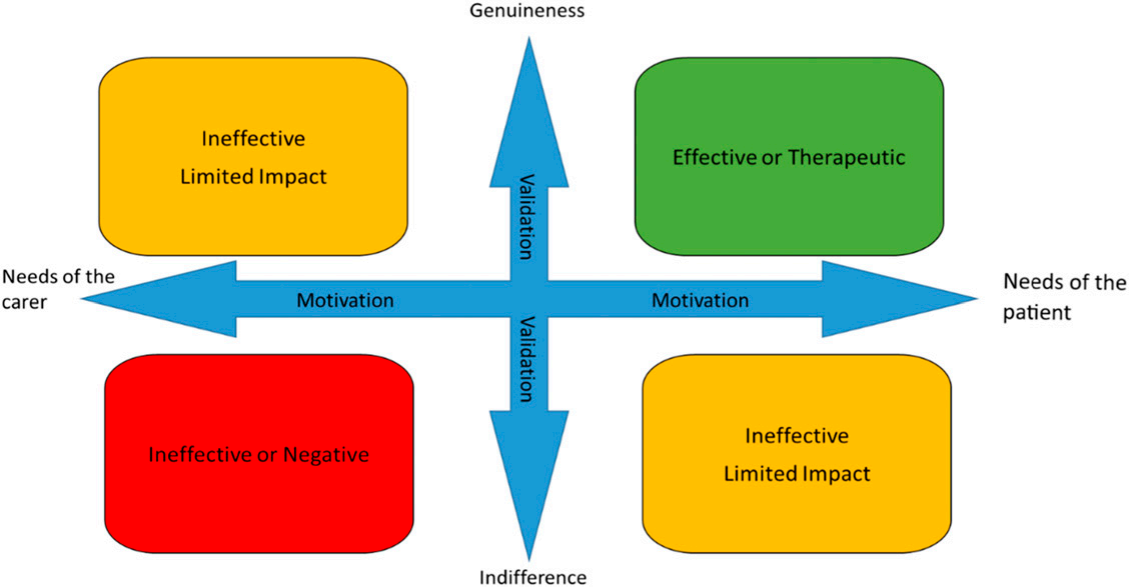
The lie ARM (Affective Reflective Model).

Motivation and validation on the part of the teller are the indicative elements as to whether the lie is likely to be effective or therapeutic. When the teller demonstrates empathy and understanding with the aim of meeting the needs of the patient, the result of the interaction was always positive. This was true even for people with advanced dementia, as the ability to experience and feel emotion remains long after cognitive reasoning has diminished.^25^ These affective domains were used to develop the Lie ARM (Affective Reflection Model) Figure 2.

## Lie ARM

### Discussion

Kitwood^33^ argues that lie telling is part of a malignant social psychology but he also identifies that there is a necessity to engage with the psychology of the person with dementia.^34^ He also emphasised the importance of personhood. Given that humans are narrative beings and an individual’s story is key to their identity and personhood,^35^ when a person with dementia tells their version of their story, it becomes essential to go along with that person’s truth to maintain both personhood and dignity. This is likely to involve telling lies that can be categorised into one of the themes identified in the Taxonomy.

One of the most significant findings of the research is the importance of the affective domains of validation, genuineness and motivation when communicating with a person with dementia. The impact these domains had on interactions was apparent during every interaction regardless of the lie being told and were the main influencers with regard to patient outcomes. Each of these domains are covered to varying degrees in the literature but have not been previously explored in combination when considering lie telling.

Within the literature there is a consensus that validation is an important and positive strategy for communicating on a verbal and emotional level, with people with dementia.^36,37^ However, the proponents of validation as a therapy argue that it is based on truth. Whilst validation is an essential part of communicating with a person with dementia, the findings of this study demonstrate that validation is often based on untruths, that is, lies. In order to be able to use validation techniques effectively, healthcare professionals need to be able to demonstrate empathy and genuineness. Validation was chosen as part of the model, rather than empathy because the significant action on behalf of the teller is demonstrating their empathy by using validation. A person can feel empathy but not necessarily demonstrate it. It is the demonstration or communication of the emotion that was significant in the observed interactions.

Genuineness and lying initially appears to present a level of dissonance, which would be the case if the model was addressing content. However, the Lie ARM specifically looks at the affective elements of the interaction. A person can be genuine in terms of their affective domain, even if what they are saying, is not representative of their own truth. It is that genuine empathy for the person with dementia that makes the validation effective. If a person interacts with indifference, it will not have a positive effect on the receiver, as was demonstrated in the study.

The motivation for an interaction involving telling a lie is key in relation to whether it is likely to be successful. If the motivation is to meet the needs of the patient, it is more likely to be successful or generate the required response from the receiver. If the motivation is to meet the needs of the nurse or carer, it is far less likely to be effective. For example, if a healthcare professional tells a lie because they are busy and need to complete a series of interventions quickly, it is unlikely to be positive because the personal motivation is likely to reduce the genuineness that the lie is said with and will not adequately validate the patient’s emotions. If the lie is said because the goal of the healthcare professional is to meet the needs of the patient to the best of their ability, they will be genuine in their interaction and will validate the patient’s emotions. Motivation was also highlighted as a key factor in telling a ‘good lie’ in a study by Casey et al.,^38^ who identified motivation as being important not only to carers, but by people with memory problems who at some point may well be the receivers of untruths. This study found that reducing emotional distress overrode any other ethical concerns and made lie telling justifiable.

Healthcare professionals, working with people with moderate to severe dementia are encouraged to reflect on their interactions using both the taxonomy and the Lie ARM. Initially, they should consider where their interaction sits, in terms of the taxonomy. This helps them to consider the motivation for the interaction; was it deliberate or spontaneous. They should then plot the interaction on the Lie ARM and consider why it was effective or not, helping to develop a more structured approach to their reflection.

## Limitations and strengths

There is a limitation of qualitative research with regard to the non-generalisability of the findings. However, in this study, every precaution was taken to be transparent and reflexive so that the reader can make a judgement about the researchers influence on the study. The outputs, that is, the taxonomy and the Lie ARM will have their validity tested in other environments in future, planned studies.

People with dementia themselves were not involved in the development of the study. Consulting service users, patients and the public has been shown to be beneficial to the development of research studies.^39^ It would have therefore been useful to consider the views and opinions of people with dementia and their families when developing and designing the study. The real strength of this study is in the ethnographic methodology which brings a new lens to the area of lie telling, by observing and recording what is happening in specific areas of practice.

## Conclusion

The issue of lie telling continues to present ethical dilemmas in everyday practice. This study has demonstrated that lie telling may be much more common than previously thought and, in many ways, more complicated. Planned and spontaneous lies have a place in nursing care, but healthcare professionals need to be much more aware of when and why they are using them, to ensure that the needs of the patients are always the priority. The Taxonomy of Lies and the Lie ARM are potentially useful tools to support deeper levels of reflection in practice. Whilst truth should always be the starting point, lying can be a useful, person-centred intervention that helps to support personhood in people with moderate to severe dementia.
